# A bivalent protein targeting glycans and HR1 domain in spike protein potently inhibited infection of SARS-CoV-2 and other human coronaviruses

**DOI:** 10.1186/s13578-021-00638-w

**Published:** 2021-07-08

**Authors:** Yanxing Cai, Wei Xu, Jiayi Tang, Najing Cao, Qiaoshuai Lan, Lu Lu, Shibo Jiang

**Affiliations:** Key Laboratory of Medical Molecular Virology (MOE/NHC/CAMS), School of Basic Medical Sciences and Biosafety Level 3 Laboratory, Shanghai Institute of Infectious Disease and Biosecurity, Fudan University, Shanghai, 200032 China

**Keywords:** GRFT, EK1, GL25E, SARS-CoV-2, Coronavirus

## Abstract

**Background:**

Our previous studies have shown that combining the antiviral lectin GRFT and the pan-CoV fusion inhibitory peptide EK1 results in highly potent inhibitory activity against SARS-CoV-2 infection. In this study, we aimed to design and construct a bivalent protein consisting of GRFT and EK1 components and evaluate its inhibitory activity and mechanism of action against infection by SARS-CoV-2 and its mutants, as well as other human coronaviruses (HCoVs).

**Methods:**

The bivalent proteins were expressed in *E. coli* and purified with Ni-NTA column. HIV backbone-based pseudovirus (PsV) infection and HCoV S-mediated cell–cell fusion assays were performed to test their inhibitory activity. ELISA and Native-PAGE were conducted to illustrate the mechanism of action of these bivalent proteins. Five-day-old newborn mice were intranasally administrated with a selected bivalent protein before or after HCoV-OC43 challenge, and its protective effect was monitored for 14 days.

**Results:**

Among the three bivalent proteins purified, GL25E exhibited the most potent inhibitory activity against infection of SARS-CoV-2 PsVs expressing wild-type and mutated S protein. GL25E was significantly more effective than GRFT and EK1 alone in inhibiting HCoV S-mediated cell–cell fusion, as well as infection by SARS-CoV-2 and other HCoVs, including SARS-CoV, MERS-CoV, HCoV-229E, HCoV-NL63 and HCoV-OC43. GL25E could inhibit authentic SASR-CoV-2, HCoV-OC43 and HCoV-229E infection in vitro and prevent newborn mice from authentic HCoV-OC43 infection in vivo. GL25E could bind to glycans in the S1 subunit and HR1 in the S2 subunit in S protein, showing a mechanism of action similar to that of GRFT and EK1 alone.

**Conclusions:**

Since GL25E showed highly potent and broad-spectrum inhibitory activity against infection of SARS-CoV-2 and its mutants, as well as other HCoVs, it is a promising candidate for further development as a broad-spectrum anti-HCoV therapeutic and prophylactic to treat and prevent COVID-19 and other emerging HCoV diseases.

## Background

The pandemic of Coronavirus Disease 2019 (COVID-19) caused by severe acute respiratory syndrome coronavirus 2 (SARS-CoV-2) infection has resulted in more than 147 million confirmed cases and over 3.1 million deaths as of 25 April, 2021. Although several COVID-19 vaccines and neutralizing antibodies have been approved for emergency use to prevent and treat SARS-CoV-2 infection, the emergence of a series of SARS-CoV-2 variants with increased transmissibility and decreased sensitivity to neutralizing antibodies has posed more severe threats to global public health and economy [1]. Therefore, the development of highly effective and broad-spectrum COVID-19 therapeutics and prophylactics is urgently needed [2].

To enter target cells, SARS-CoV-2 binds to its receptor ACE2 on the host cell through the receptor-binding domain (RBD) in S1 subunit of spike (S) protein [3]. Such binding triggers conformation changes in the S2 subunit of S protein, resulting in the formation of a six-helix bundle (6-HB) between the heptad repeat 1 and 2 (HR1 and HR2) domains, thus bringing viral and target cell membranes together for fusion [4]. Therefore, both S1 and S2 subunits can serve as important targets for the development of SARS-CoV-2 fusion and entry inhibitors [5]. The sequence of S2 subunit is more conserved than that of S1 subunit, making it a better target for developing broad-spectrum SARS-CoV-2 entry inhibitors [6].

In our previous studies, we have identified two pan-CoV fusion and entry inhibitors, EK1 and griffithsin (GRFT) [6–8]. EK1, a pan-CoV fusion inhibitor, could effectively inhibit infection by SARS-CoV-2 and other HCoVs by binding to HR1 of these HCoVs and blocking 6-HB formation and, hence, membrane fusion [4, 9]. GRFT is an antiviral lectin derived from the red alga *Griffithsia* sp. and consists of 121 amino acids [10] able to interact with glycans displayed on SARS-CoV-2 S protein and inhibit SARS-CoV-2 infection without blocking the binding of RBD to ACE2 [7]. Moreover, the combination of EK1 and GRFT shows potent synergistic inhibitory effect on SARS-CoV-2 infection. Based on the above findings, we hypothesized that a recombinant protein capable of binding to both HR1 and glycans in S protein of SARS-CoV-2 might exhibit more potent inhibitory activity against SARS-CoV-2 infection than either GRFT or EK1 alone. To this end, we designed three bivalent proteins (GRFT-L15-EK1, GRFT-L25-EK1 and GRFT-L35-EK1) by covalently linking GRFT and EK1 with a 15-, 25- or 35-mer linker, respectively. We found that GRFT-L25-EK1 (GL25E), which has a 25-mer linker (GGGGS)_5_, exhibited the most potent anti-SARS-CoV-2 activity with half maximal inhibitory concentration (IC_50_) at low nM concentration, much more effective than GRFT and EK1 alone. Further studies have shown that GL25E can bind to both glycans in RBD and S1 subunit and to HR1 in S2 subunit in S protein of SARS-CoV-2. GL25E is also much more effective than GRFT and EK1 alone against infection by SARS-CoV-2 pseudoviruses (PsVs) with mutations in RBD and by other HCoVs. Therefore, it is a promising candidate for further development as a therapeutic and prophylactic to treat and prevent COVID-19 and other emerging HCoV diseases.

## Methods

### Cells, viruses, proteins and peptides

HuH-7 and Caco-2 cells were obtained from the Cell Bank of the Chinese Academy of Science. HEK-293T (CRL-3216™) cells and RD (CCL-136™) were obtained from the American Type Culture Collection (ATCC). All these cells were cultured in DMEM supplemented with 10% FBS, 100 U/ml penicillin and 100 mg/ml streptomycin. HCoV-OC43 (VR-1558) and HCoV-229E (VR-740) were obtained from ATCC and amplified in HCT-8 and HuH-7 cells, respectively. His-tagged human ACE2, Fc-tagged RBD and Fc-tagged S1 subunit were obtained from Kactus Biosystems. EK1 peptide was synthesized by KareBay Biochem with a purity of > 95%.

### The production, purification and identification of GRFT, GL15E, GL25E and GL35E

Optimized gene sequences encoding GRFT, GL15E, GL25E and GL35E with *E. coli* codon preference were synthesized by the Beijing Genomics Institution and constructed into pET-28a. Recombinant plasmids were transformed into *E. coli* BL21 (DE3). The transformed BL21 (DE3) was cultured in the presence of 50 μg/ml of kanamycin at 37℃ until the culture density reached 0.5 at OD600. The culture was placed on ice for 15 min and then incubated in the presence of 1 mM IPTG at 16℃ with shaking (180 rpm) for 20 h. The cells were harvested at 8,000 rpm for 3 min. The recombinant proteins were purified with Ni–NTA column and further confirmed by SDS-PAGE and Western blot as previously described [11].

### Production of pseudotyped coronaviruses

HCoV PsVs were produced as previously described [6, 9]. Briefly, HEK-293T cells were co-transfected with pNL4-3.Luc.R-E- and HCoV S protein-expressing plasmids (including pcDNA3.1-SARS-CoV-2-S, pcDNA3.1-SARS-CoV-S, pcDNA3.1-MERS-CoV-S, pcDNA3.1-HCoV-OC43-S, pcDNA3.1-HCoV-229E-S and pcDNA3.1-HCoV-NL63-S). The supernatants containing PsVs were harvested 72 h after transfection and stored at −80℃.

### Inhibition of HCoV PsV infection

PsV infection inhibition assay was performed as previously described [9]. Briefly, target cells (RD cells for HCoV-OC43 and HuH-7 cells for other coronaviruses) were seeded into a 96-well culture plate one day prior to infection. Inhibitors at the indicated concentrations were mixed with PsVs and incubated at 37 ℃ for 30 min. The mixture was added to target cells and incubated for 12 h. The mixture was then removed, and target cells were cultured in fresh DMEM for 48 h. Luciferase assay was performed to test the inhibitory activity against coronavirus PsV infection.

### Inhibition of HCoV S-mediated cell–cell fusion

Inhibition of HCoV S-mediated cell–cell fusion assay was conducted as previously described [9]. Briefly, HEK-293T cells were transfected with plasmids expressing HCoV S protein/GFP. After culturing for 48 h, HEK-293T cells expressing HCoV S protein were prepared as a single-cell suspension and co-incubated with inhibitors at the indicated concentrations at 37 ℃ for 1 h. The mixture was added into target cells and co-incubated. After observing formation of the cell–cell fusion, four fields were randomly selected to calculate the fusion rate.

### Inhibition of cytopathic effect mediated by authentic HCoV infection

Target cells (Vero-E6 cells for SARS-CoV-2, RD cells for HCoV-OC43 and HuH-7 cells for HCoV-229E) were seeded into a 96-well culture plate one day prior to infection. Inhibitors at the indicated concentrations were mixed with SARS-CoV-2 (SARS-CoV-2/SH01/human/2020/CHN, 50 plaque-forming units), HCoV-OC43 (VR-1558, 100 TCID_50_) or HCoV-229E (VR-740, 100 TCID_50_) and co-incubated for 1 h at 37 ℃. The mixture was added into target cells and incubated for 1 h (for SARS-CoV-2) or 12 h (for HCoV-OC43 and HCoV-229E). The mixture was removed, and cells were cultured for 72 h. The inhibitory effect of GL25E on authentic HCoV infection was measured as previously described [9].

### The binding of GL25E to S1 subunit and RBD

S1 subunit or RBD (2 μg/ml) was added into a 96-well polystyrene plate and incubated at 37 ℃ for 2 h. The plate was washed with PBST and blocked with 2% gelatin at 37 ℃ for 2 h. After washing with PBST, the plate was incubated with GL25E at the indicated concentrations in the presence or absence of mannose at 37 ℃ for 1 h. The plate was washed with PBST 3 times, and HRP-conjugated anti-6His antibody (1:3000, ProteinTech) was added into plate. After incubation of 1 h, the plate was washed with PBST 4 times, and TMB was added into plate. The reaction was stopped and measured at 450 nm.

### Inhibition of 6-HB formation by GL25E

The 6-HB formation inhibition assay was performed as previously described [12]. Briefly, HR1P was dissolved in PBS in the presence or absence of GL25E at the indicated concentrations at 25 ℃ for 30 min. HR2P was added into the mixture and co-incubated at 25 ℃ for 30 min. The mixture was subjected to Native-PAGE, followed by staining the gel with Coomassie blue and imaging.

### In vivo inhibition of authentic HCoV-OC43 infection

Newborn mice at 5 days were obtained from pregnant BABL/c mice purchased from the Shanghai Laboratory Animal Research Center. To test the in vivo prophylactic activity of GL25E, PBS or GL25E at either high dose (2 mg/kg) or low dose (0.4 mg/kg) was intranasally administered 0.5 h prior to authentic HCoV-OC43 challenge (100 TCID_50_). To test the in vivo therapeutic activity of GL25E, PBS or GL25E at either high dose (4 mg/kg) or low dose (0.8 mg/kg) was intranasally administered 0.5 h after authentic HCoV-OC43 challenge (100 TCID_50_). To investigate the effect of administration time on the in vivo inhibitory activity of GL25E, newborn mice were intranasally treated with GL25E (4 mg/kg) 2 h (pre-2 h), 24 h (pre-24 h) before or 2 h (post-2 h), 24 h (post-24 h) after virus challenge, respectively. Body weight and survival rate were monitored daily for 14 days.

### Statistical analysis

The IC_50_ values of all inhibitors were calculated using the CalcuSyn program [13] (kindly provided by Dr. T. C. Chou). The statistical analyses were performed with GraphPad Prism software and significant difference between two groups was analyzed using the unpaired two-tailed Student’s -*t* test.

## Results

### Design, expression and identification of the bivalent proteins

**Fig. 1:**
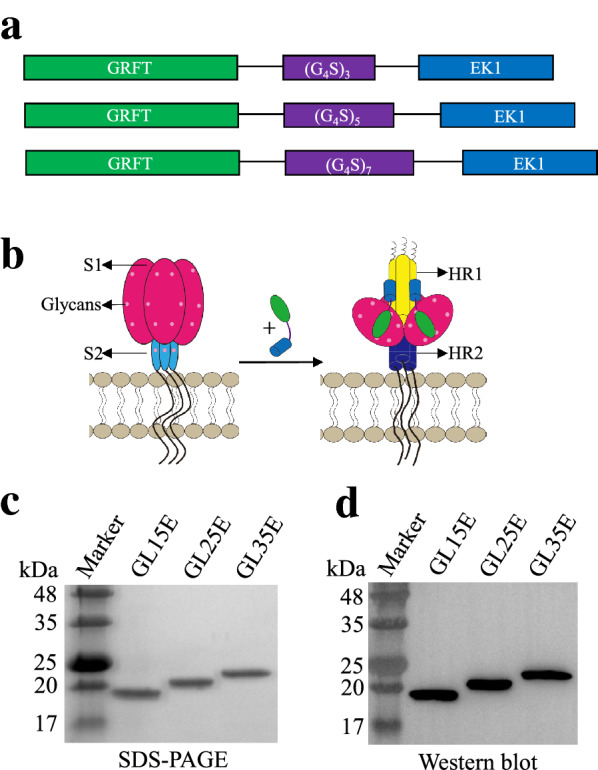
Design, expression and identification of GL15E, GL25E and GL35E. **a** The design of GL15E, GL25E and GL35E. **b** Schematic view of the binding between GRFT and EK1 components in the bivalent protein to the glycans in S1 subunit and HR1 in S2 subunit of S protein of SARS-CoV-2 or other HCoVs. Identification of three bivalent proteins by SDS-PAGE (**c**) and Western blot (**d**)

To understand the influence of linker length on the inhibitory activity of the recombinant bivalent proteins, we designed and constructed three pET-28a recombinant plasmids encoding three recombinant bivalent proteins, GRFT-L15-EK1 (GL15E), GRFT-L25-EK1 (GL25E), and GRFT-L35-EK1 (GL35E), containing linkers L15 (GGGGS)_3_, L25 (GGGGS)_5_, and L35 (GGGGS)_7_ between the GRFT and EK1 components, respectively (Fig. 1a and b). The recombinant bivalent proteins were expressed in *E. coli*. After purification by Ni–NTA column, the recombinant proteins were subjected to SDS-PAGE and Western blot analysis. As shown in Fig. 1c and d, the three recombinant proteins migrated near the expected position on the gels (GL15E: ~ 20 kDa; GL25E, ~ 21 kDa; GL35E: ~ 22 kDa).

### The bivalent proteins exhibited more potent inhibitory activity against wild-type pseudotyped SARS-CoV-2 infection

**Fig. 2:**
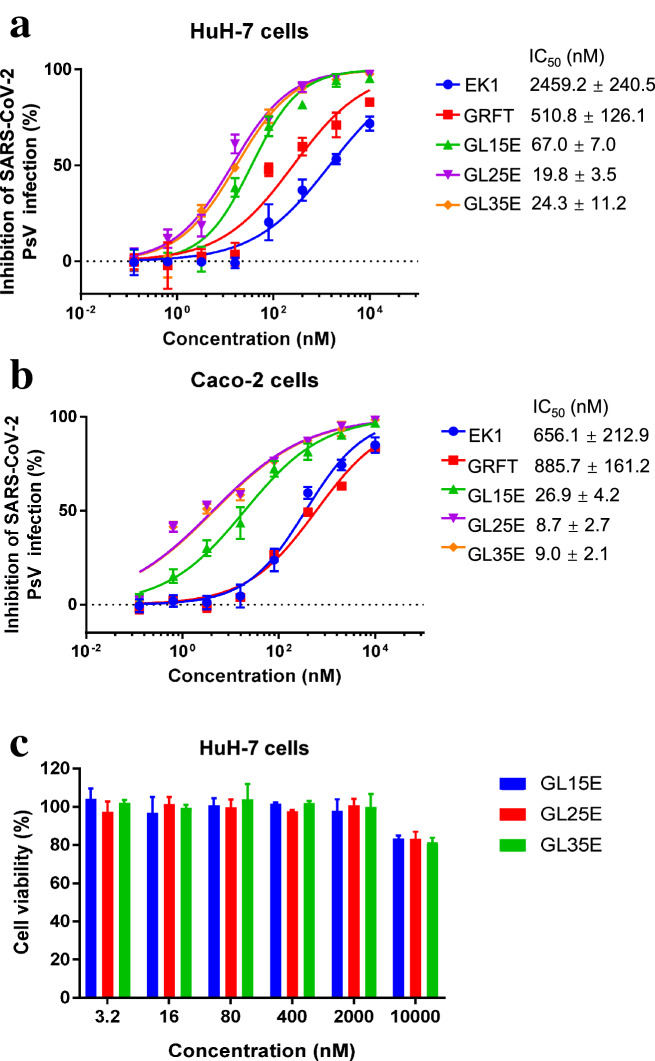
The effect of bivalent proteins on SARS-CoV-2 PsV infection and their cytotoxicity to HuH-7 cells. The inhibitory activity of GL15E, GL25E and GL35E on SARS-CoV-2 PsV infection in HuH-7 cells (**a**) or Caco-2 cells (**b**). The effect of GL15E, GL25E and GL35E on the viability of HuH-7 cells (**c**). Each sample was tested in triplicate, and the experiment was repeated at least twice. Data from a representative experiment are presented as means ± SD

We then utilized HIV backbone-based SARS-CoV-2 pseudovirus (PsV) to assess the inhibitory activity of GL15E, GL25E and GL35E against the infection of SARS-CoV-2 PsV expressing wild-type S protein in HuH-7 cells. As shown in Fig. 2a, GRFT and EK1 tested alone showed effective inhibitory activity against SARS-CoV-2 PsV infection with IC_50_ of 511 and 2459 nM, respectively (Fig. 2a). However, GL15E, GL25E and GL35E had much improved inhibitory activity against SARS-CoV-2 PsV infection with IC_50_ of 67, 20 and 24 nM, respectively (Fig. 2a). The inhibitory activity of GL25E was about 25- and 123-fold more potent than that of GRFT and EK1 alone, respectively.

GL15E, GL25E and GL35E were also effective in inhibiting infection of SARS-CoV-2 PsV infection in Caco-2 cells, a human intestinal cell line that could also be infected by SARS-CoV-2 with IC_50_ of 27, 9 and 9 nM, respectively (Fig. 2b). The inhibitory activity of GL25E on SARS-CoV-2 PsV infection was about 74- and 101-fold more potent than that of EK1 and GRFT alone.

To rule out the influence of cytotoxicity on the inhibitory activity, we then tested the effect of the three bivalent proteins on cell viability by treating HuH-7 cells with GL15E, GL25E and GL35E at different concentrations for 48 h. As shown in Fig. 2c, the half maximal cytotoxicity concentration (CC_50_) of the bivalent proteins on HuH-7 cells was more than 10,000 nM, which was much higher than the IC_50_ of the bivalent proteins against SARS-CoV-2 PsV infection, confirming that the inhibitory activity of the bivalent proteins cannot be attributed to their cytotoxicity. The above results suggest that the best anti-SARS-CoV activity of bivalent protein GL25E can likely be attributed to the linker between GRFT and EK1 having the needed length to allow both GRFT and EK1 components in the recombinant protein to reach and interact with their corresponding targets in S1 and S2 subunits, respectively. It was concluded that GL25E has much higher potency in inhibiting SARS-CoV-2 infection than either GRFT or EK1 alone, possibly by the synergism of GRFT and EK1 acting together in recombinant bivalent protein GL25E.

### GL25E was highly effective in inhibiting infection by SARS-CoV-2 PsVs with mutation in S protein.

**Fig. 3:**
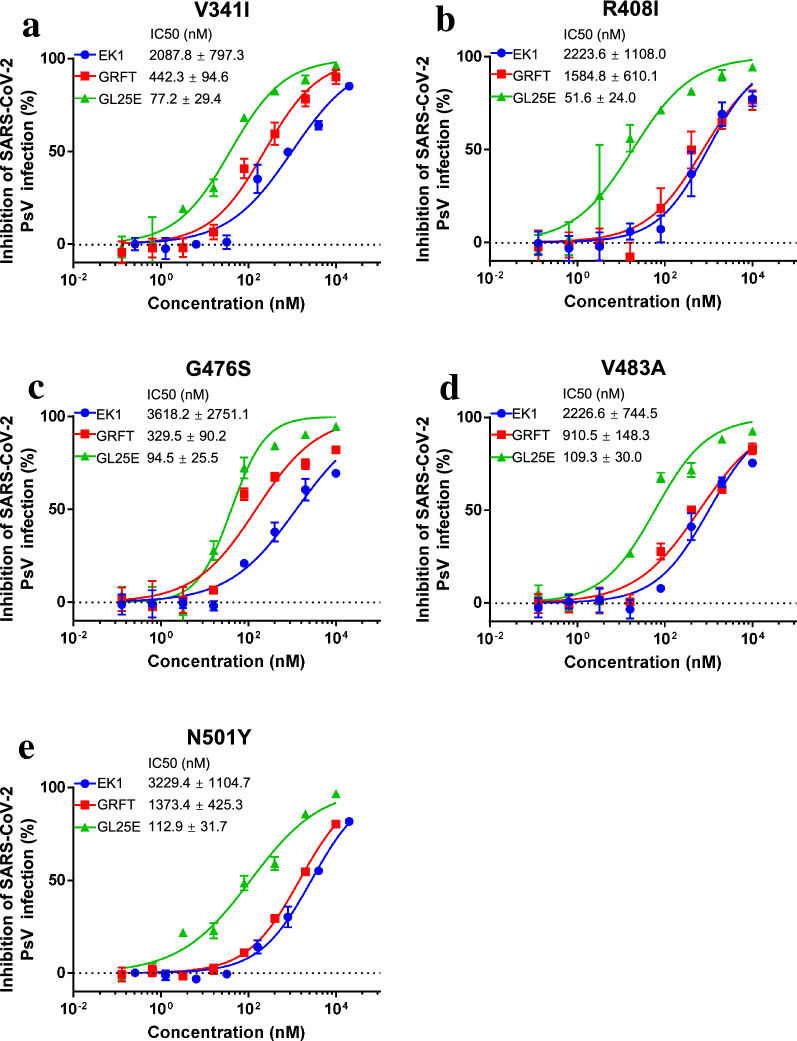
The effect of GL25E on infection of SARS-CoV-2 PsVs with mutations in RBD of SARS-CoV-2. Inhibition of GL25E on infection of SARS-CoV-2 PsV with mutation in RBD of S protein, including V341I (**a**), R408I (**b**), G476S (**c**), V483A (**d**) and N501Y (**e**). Each sample was tested in triplicate, and the experiment was repeated at least twice. Data from a representative experiment are presented as means ± SD

It has been reported that SARS-CoV-2 has continuously mutated in its S protein, resulting in enhanced viral transmissibility and increased viral resistance to neutralizing antibodies and entry inhibitors [14]. To investigate whether GL25E could inhibit the infection of SARS-CoV-2 mutants, we generated 5 SARS-CoV-2 PsVs with single amino acid mutation in S protein, including V341I, R408I, G476S, V483A and N501Y, and used them to test the effect of these mutations on the inhibitory activity of GL25E on SARS-CoV-2 PsV infection. As shown in Fig. 3a–f, GL25E could also effectively inhibit infection of SARS-CoV-2 PsVs having V341I, R408I, G476S, V483A or N501Y mutation with IC_50_ of 83, 52, 95, 109, and 113 nM, respectively, which, again, were values more potent than those of GRFT and EK1 alone. These results suggest that GL25E can also effectively inhibit infection by SARS-CoV-2 variants with mutations in its S protein.

### GL25E could recognize glycans on S1 subunit, particularly RBD, and inhibit SARS-CoV-2 S-mediated cell–cell fusion by interfering with 6-HB formation

**Fig. 4:**
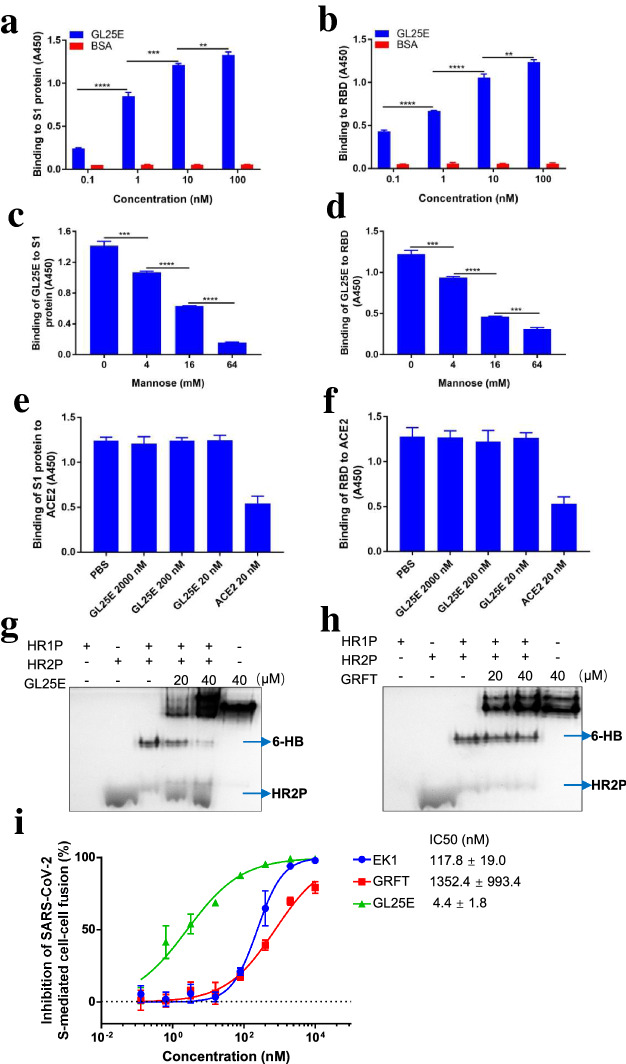
The mechanism of action of GL25E in inhibiting SARS-CoV-2 infection. The binding of GL25E to S1 subunit (**a**) and RBD (**b**) as measured by ELISA. The effect of mannose on the binding of GL25E to S1 subunit (**c**) and RBD (**d**). The effect of GL25E on the binding of S1 subunit (**e**) and RBD (**f**) to ACE2. The effect of GL25E (**g**) and GRFT (**h**) on 6-HB formation. The inhibition of GL25E on SARS-CoV-2 S-mediated cell–cell fusion (**i**). Each sample was tested in triplicate and the data were presented as means ± SD. Significant difference between two groups was analyzed using the unpaired two-tailed Student’s -*t* test. **P < 0.01; ***P < 0.001; ****P < 0.0001

To illustrate the antiviral mechanism of GL25E, we first investigated whether GL25E retained the bioactivity of GRFT to bind to S1 subunit and RBD. As shown in Fig. 4a and b, GL25E could bind to S1 subunit and RBD in a dose-dependent manner. Next, we investigated whether GL25E bound to S1 subunit and RBD in a carbohydrate-dependent manner. As shown in Fig. 4c and d, mannose could block the binding of GL25E to S1 subunit and RBD in a dose-dependent manner, indicating that GL25E possessed the ability to bind to the glycans displayed on the S1 subunit and RBD. However, the binding of S1 subunit or RBD to ACE2 was not inhibited by GL25E (Fig. 4e and f). The above results are consistent with the results from the experiment for testing GRFT alone [7].

We then investigated whether GL25E, like EK1, could interact with HR1 in SARS-CoV-2 S2 subunit to interfere with 6-HB formation. Native-PAGE was performed to determine 6-HB formation in the presence and absence of GL25E. As shown Fig. 4g and h, GL25E could significantly inhibit 6-HB formation as indicated by the decreased density of 6-HB band and increased density of HR2P band, respectively, whereas GRFT alone at the indicated concentrations showed no significant inhibition on 6-HB formation. Considering that 6-HB formation plays a key role in mediating fusion between viral and host cell membranes, we speculated that GL25E may interact with HR1 of the viral SARS-CoV-2 S protein to block the fusion between SARS-CoV-2 and host cells. We thus performed a SARS-CoV-2 S-mediated cell–cell fusion assay and found that GL25E was very effective in inhibiting SARS-CoV-2 S-mediated cell–cell fusion in a dose-dependent manner with IC_50_ of 4 nM, about 307- and 27-fold more potent than that of GRFT alone (IC_50_ = 1352 nM) and 27-fold greater than that of EK1 alone (IC_50_ = 118 nM), respectively (Fig. 4i).

These results indicated that GL25E could bind, through its GRFT component, to glycans displayed on S1 subunit and RBD and to HR1 in the S2 subunit of SARS-CoV-2 S protein via its EK1 component, thus blocking 6-HB formation and inhibiting the fusion between viral and host cell membranes.

### GL25E showed broad inhibitory activity against S-mediated cell–cell fusion and pseudovirus infection of other HCoVs.

**Fig. 5:**
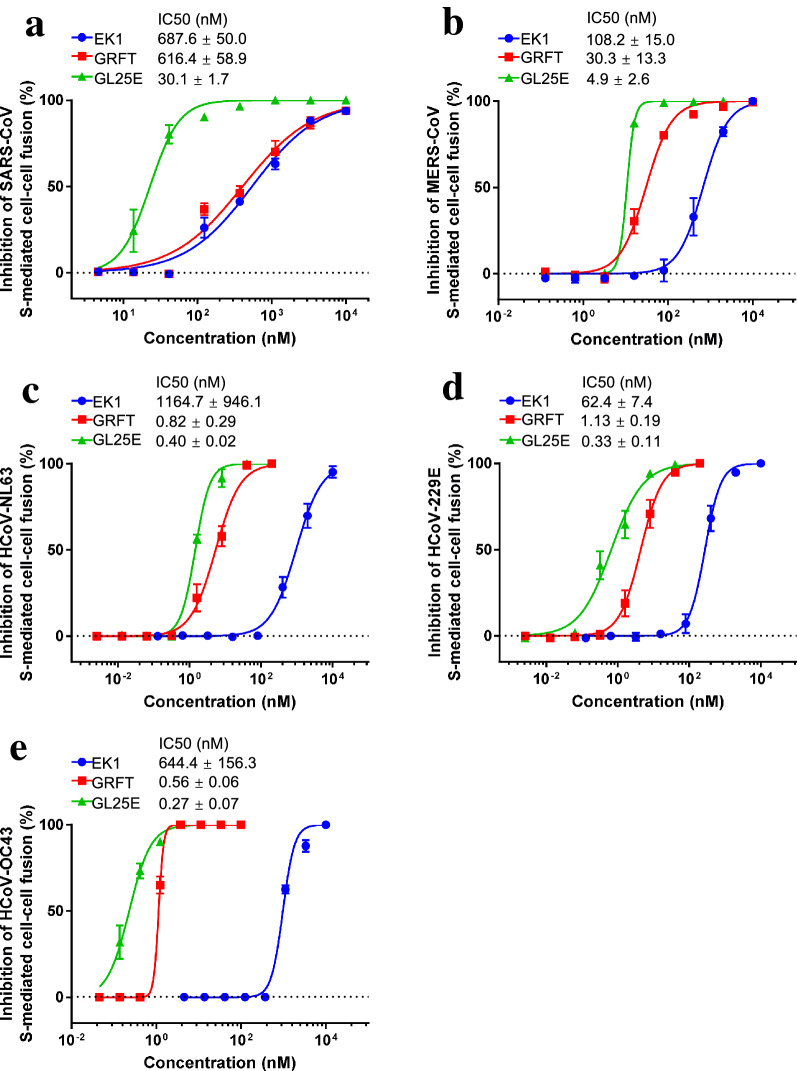
Inhibition of GL25E on cell–cell fusion medicated by S protein of HCoVs. Inhibition of GL25E on cell–cell fusion mediated by S protein of SARS-CoV (**a**), MERS-CoV (**b**), HCoV-NL63 (**c**), HCoV-229E (**d**) and HCoV-OC43 (**e**). Each sample was tested in triplicate, and the experiment was repeated at least twice. Data from a representative experiment are presented as means ± SD

**Fig. 6:**
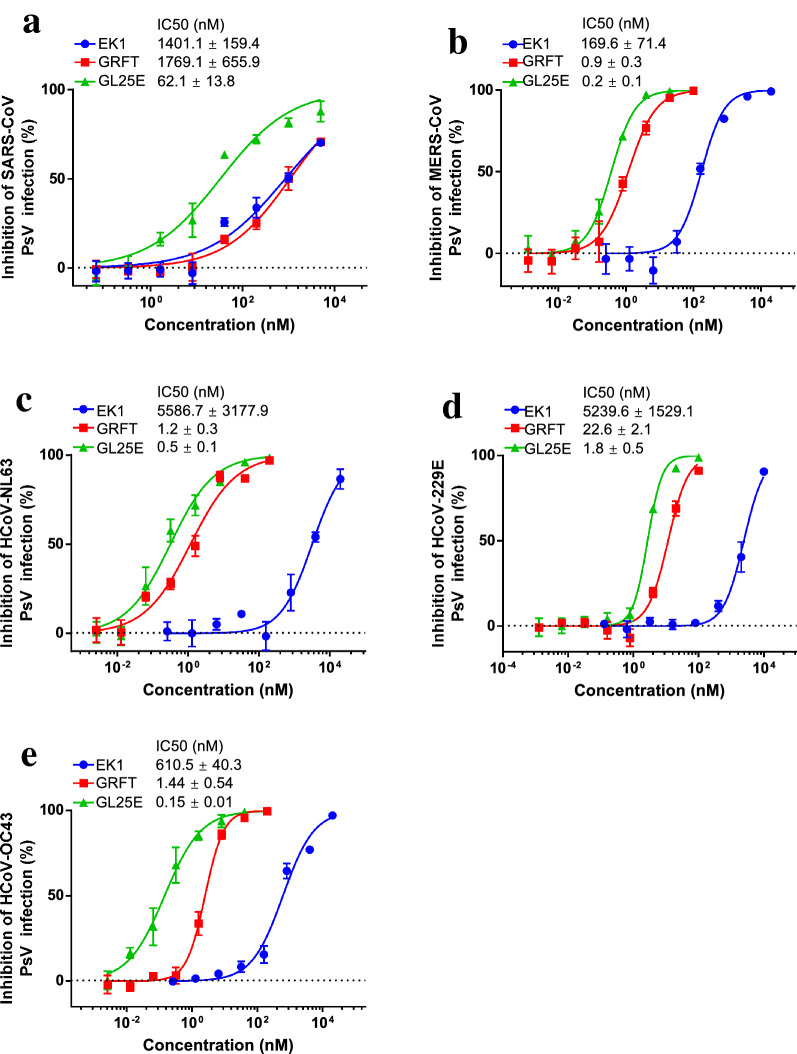
Inhibition of GL25E on HCoV PsV infection. The inhibition of GL25E on infection by the pseudotyped SARS-CoV (**a**), MERS-CoV (**b**), HCoV-NL63 (**c**), HCoV-229E (**d**) and HCoV-OC43 (**e**). Each sample was tested in triplicate, and the experiment was repeated at least twice. Data from a representative experiment are presented as means ± SD

Given that our previous studies have shown that both GRFT and EK1 alone exhibit broad-spectrum anti-HCoV activity, we then investigated whether GL25E could also effectively inhibit cell–cell fusion mediated by S protein of divergent HCoVs, including SARS-CoV, MERS-CoV, HCoV-229E, HCoV-NL63 and HCoV-OC43. As shown in Fig. 5, GL25E was highly effective in inhibiting S-mediated cell–cell fusion of each listed HCoV in a dose-dependent manner with IC_50_ ranging from 0.3 to 30 nM, which was much more potent than that of GRFT alone (IC_50_ ranging from 0.56 to 616 nM) and that of EK1 alone (IC_50_ ranging from 62 to 1,165 nM). We then assessed the inhibitory activity of GL25E against infection of divergent PsVs expressing S proteins of SARS-CoV, MERS-CoV, HCoV-NL63, HCoV-229E and HCoV-OC43. As shown in Fig. 6, GL25E was able to inhibit infection of these pseudotyped HCoVs in a dose-dependent manner with IC_50_ ranging from 0.15 to 62 nM, which was more effective than that of GRFT alone (IC_50_ ranging from 0.9 to 1769 nM) and that of EK1 alone (IC_50_ ranging from 170 to 5587 nM).

Overall, these results suggest that GL25E possesses broad-spectrum inhibitory activity against HCoV infection and that it is more potent in inhibiting HCoV S-mediated cell–cell fusion and PsV infection than GRFT and EK1 alone.

### GL25E could inhibit authentic HCoV infection in vitro and protect mice from HCoV-OC43 infection

**Fig. 7:**
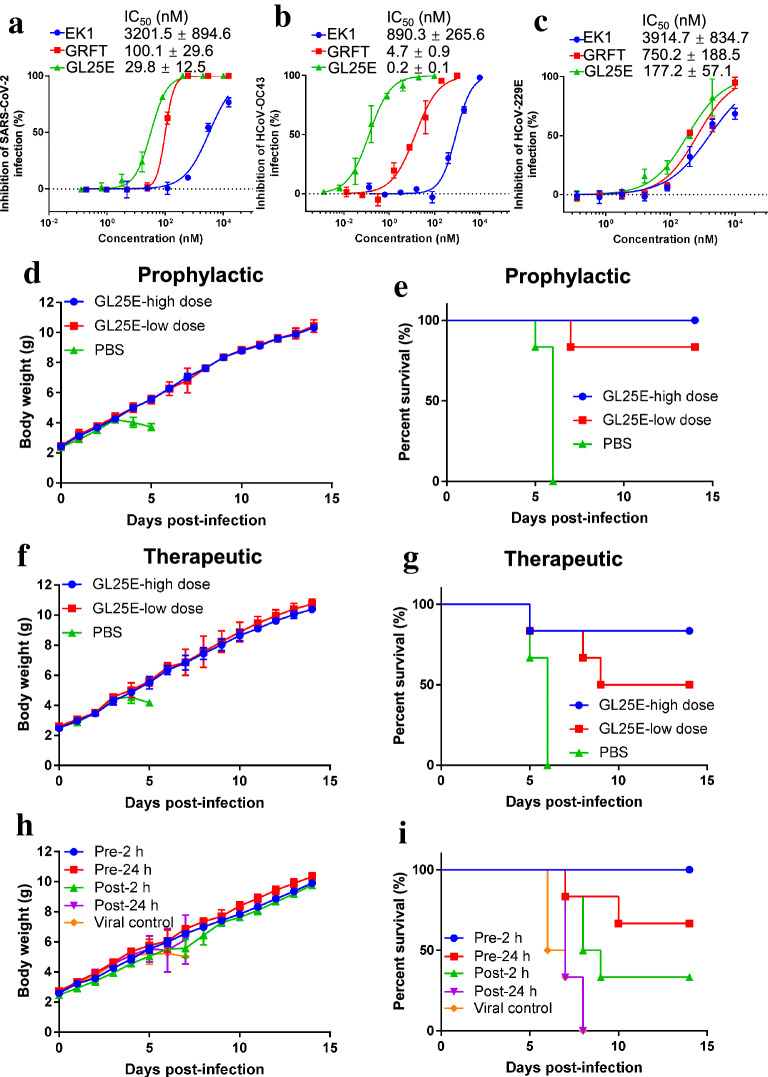
In vitro and in vivo inhibitory activity of GL25E against authentic HCoV infection. Inhibition of GL25E on in vitro infection by authentic SARS-CoV-2 (**a**), HCoV-OC43 (**b**) and HCoV-229E (**c**). Inhibition of GL25E on in vivo infection by authentic HCoV-OC43, as shown by the changes of body weight (**d**) and survival rate (**e**) of mice treated with PBS and GL25E 0.5 h prior to viral challenge, or the changes of body weight (**f**) and survival rate (**g**) of mice treated with PBS and GL25E 0.5 h after HCoV-OC43 challenge, or the changes of body weight (**h**) and survival rate (**i**) of mice treated with GL25E at indicated time before or after viral challenge. Each sample was tested in triplicate, and the experiment was repeated at least twice. Data from a representative experiment are presented as means ± SD

Subsequently, we evaluated the inhibitory activity of GL25E against infection of authentic HCoVs, including SARS-CoV-2, HCoV-OC43 and HCoV-229E. As shown in Fig. 7a, GL25E significantly inhibited authentic SARS-CoV-2 infection in a dose-dependent manner with IC_50_ of 29.8 nM, about 3.4- and 107.4-fold more potent than that of GRFT alone (IC_50_ = 100 nM) and EK1 alone (IC_50_ = 3201 nM), respectively. The cytopathic effect mediated by authentic HCoV-OC43 infection was also significantly inhibited by GL25E with IC_50_ of 0.2 nM, about 23.5- and 4451-fold more potent than that of GRFT alone (IC_50_ = 4.7 nM) and EK1 alone (IC_50_ = 890 nM), respectively (Fig. 7b). Similarly, GL25E was also effective in inhibiting the cytopathic effect mediated by authentic HCoV-229E infection with IC_50_ of 177 nM, which was about 4- and 22-fold more potent than that of GRFT alone (IC_50_ = 750 nM) and EK1 alone (IC_50_ = 3915 nM), respectively (Fig. 7c).

Next, we used an HCoV-OC43-infected mouse model to evaluate the in vivo antiviral activity of GL25E. We first detected the potential prophylactic effect of GL25E by intranasally treating newborn mice with GL25E (PBS as a control) 0.5 h before challenging the mice with 100 TCID_50_ HCoV-OC43. Mice in the PBS control group began to lose weight 4 days post-infection, and all of them died at day 6 post-infection (Fig. 7d and e). In contrast, mice treated with GL25E at high dose or low dose showed no significant loss of weight and exhibited 100% and 83% survival rate, respectively. We then investigated the therapeutic effect of GL25E by intranasally treating newborn mice with GL25E (PBS as a control) 0.5 h after challenging the mice with 100 TCID_50_ HCoV-OC43. Similarly, mice treated with GL25E at high or low dose showed no remarkable loss of weight and exhibited 83% or 50% survival rate, respectively, while all mice in the PBS-treated group died at day 6 days post-infection (Fig. 7f and g).

Subsequently, we investigated the effect of administration time on the in vivo activity of GL25E. As shown in Fig. 7h and i, no significant loss of weight was observed in groups of pre-2 h, pre-24 h and post-2 h with 100%, 67% and 33% survival rate, respectively, while all mice in post-24 h and viral control groups died at 8 days post-infection, indicating that GL25E could exhibited more promising prophylactic effect than therapeutic effect. These results suggest that once the HCoV-OC43 infection in brain is established, the therapeutic effect of GL25E may be blunted or lost due to the existence of the blood–brain barrier (BBB). Therefore, it is essential to develop a nano-delivery system in the future for transportation of GL25E across the BBB, thus enhancing its therapeutic effect.

Taken together, these results indicate that GL25E exhibits potent inhibitory activity against authentic SASR-CoV-2, HCoV-OC43 and HCoV-229E infection in vitro and intranasal administration of GL25E before or after HCoV-OC43 challenge can protect mice from HCoV-OC43 infection.

## Discussion

GRFT, as one of the most potent antiviral lectins, is able to bind the terminal mannoses present in high-mannose glycans and crosslink these glycans on the surface of the envelope glycoprotein of an enveloped virus, such as HIV, resulting in the inhibition of viral fusion with and entry to the target cell. Therefore, GRFT, as a component of an antiviral microbicide gel for the prevention of sexual transmission of HIV, was tested in clinical trials [15, 16]. Most recently, we have shown that GRFT is highly effective in inhibiting infection of both pseudotyped and authentic SARS-CoV-2 and blocking its S protein-mediated cell–cell fusion [7]. It could bind strongly to the S1 subunit or RBD in the S protein of SARS-CoV-2, but did not block binding of the S1 or RBD to the receptor ACE2, suggesting that GRFT inhibits SARS-CoV-2 infection through a mechanism of action different from most SARS-CoV-2 neutralizing antibodies that neutralize SARS-CoV-2 infection by blocking interaction between viral RBD and receptor ACE2 [17].

EK1 is a modified HCoV-OC43 HR2-derived peptide, in which Glu (E) and Lys (K) are introduced to allow them to form intramolecular salt bridges for the enhancement of solubility and antiviral activity of the peptide. We have demonstrated that EK1 can bind to HR1 of SARS-CoV-2 and other HCoVs to prevent 6-HB formation, thus blocking membrane fusion and inhibiting infection by these HCoVs tested [6, 9]. Interestingly, we found that combining GRFT and EK1 resulted in strong synergistic effect against SARS-CoV-2 infection [7]. Similarly, combinational use of GRFT with several known HIV-1 entry inhibitor-based antiviral drugs, such as the CCR5 antagonist, maraviroc and the gp41 fusion inhibitor enfuvitride exhibited potent synergistic anti-HIV-1 activity, showing high potential in the development of anti-HIV microbicides [18]. Kagiampakis et al. constructed a bivalent recombinant protein by covalently linking the HIV-1 gp120-binding lectin GRFT with the HIV-1 gp41-binding peptide C37. The resultant protein, Griff37, exhibits anti-HIV-1 potency about 8- and 120-fold greater than that of griffithsin and C37 alone, respectively [19].

In this study, we have adopted this strategy to design and construct a bivalent recombinant protein with bifunctional antiviral activity by linking GRFT with EK1, in order to improve the inhibitory activity against SARS-CoV-2 infection. We found that the three bivalent recombinant proteins with different length of linker all exhibited potent inhibitory activity against SARS-CoV-2 PsV infection and its S protein-mediated cell–cell fusion, while GL25E, the bivalent protein with 25-mer linker (GRFT-L25-EK1), displayed the most potent antiviral activity against infection by the pseudotyped and authentic SARS-CoV-2 at low nM concentration, which is significantly more effective than GRFT and EK1 alone. Further studies have shown that GL25E can bind to glycans in RBD and S1 subunit in S protein of SARS-CoV-2, but it did not block the interaction between viral RBD or S1 subunit and cellular receptor ACE2, consistent with the result from the experiment for testing GRFT alone in our previous report [7], suggesting that the GRFT component in GL25E and GRFT protein share a common mechanism of action against SARS-CoV-2 infection. GL25E could effectively block 6-HB formation between the HR1- and HR2-derived peptides and potently inhibited SARS-CoV-2 S protein-mediated cell–cell fusion, indicating that the EK1 component in GL25E and EK1 peptide also have a similar mechanism of action against SARS-CoV-2 S protein-mediated membrane fusion and viral entry [4, 6].

In addition to the synergistic effect, GL25E may have several more advantages over GRFT and EK1. First, GL25E has a bigger molecular size than GRFT and EK1, and is thus expected to have longer half-life, providing stronger steric blocking effect against association between viral HR1 and HR2 to form 6-HB. Second, GL25E may have higher concentration than EK1 on the viral surface to facilitate the interaction of the EK1 component with viral HR1 to interfere with viral 6-HB formation because GL25E can bind, via its GRFT component, to the glycans on viral envelope glycoprotein. Third, compared to EK1, GL25E may more easily enter into the endosome to block endosomal membrane fusion mediated by the EK1 component in GL25E. Endosomal entry may also be facilitated by the binding of GL25E, via its GRFT component, to the glycans on viral surface glycoprotein. Most HCoVs enter into the host cell through both plasma and endosomal membrane fusion pathways [9]. EK1 peptide, which cannot bind the HR1 domain in S protein at the native or pre-fusion conformation, cannot easily penetrate the endosome in the target cell.

It should be noted that SARS-CoV-2 has been undergoing mutations continuously to date, raising the concerns that the SARS-CoV-2 variants may gain resistance to SARS-CoV-2 neutralizing antibodies and entry inhibitors [20, 21]. Particularly, several emerging variants carrying the N501Y mutation in their S protein rapidly became dominant in the world, indicating that the variants with N501Y mutation may possess a transmission advantage over the wild-type SARS-CoV-2 strains [22, 23]. Haffman et al. have reported that several SARS-CoV-2 variants with N501Y and other mutations in RBD have become resistant to the neutralizing antibodies bamlanivimab and REGN10989, while they are still highly sensitive to the inhibitory activity of our pan-CoV fusion inhibitory peptides EK1 and EK1C4 [24]. Here we found that GL25E could also effectively inhibit infection by PsVs with N501Y and other mutations in RBD of SARS-CoV-2 S protein. GL25E is also much more effective than GRFT and EK1 alone against infection by other HCoVs, including SARS-CoV, MERS-CoV, HCoV-OC43, HCoV-NL63 and HCoV-229E.

GL25E has another advantage as an anti-HCoV agent for clinical use over neutralizing antibodies by presenting a higher genetic barrier to resistance than a neutralizing antibody. The EK1 component in GL25E targets the HR1 domain in S2 subunit, which is much more conserved than the RBD in the S1 subunit targeted by most SARS-CoV-2 neutralizing antibodies. With its smaller molecular size compared to an IgG antibody, GL25E can be used in an inhalation formulation to prevent and treat SARS-CoV-2 infection.

In addition to GL25E, we have previously identified another highly potent pan-CoV fusion inhibitor, EK1C4, by conjugating cholesterol to the C-terminus of EK1 [9]. EK1C4 is as potent as GL25E in inhibiting SARS-CoV-2 infection. However, the cost for synthesis of lipopeptide EK1C4 at large scale is much higher than the production of GL25E using an E. coli expression system. Therefore, GL25E has a better potential than the neutralizing antibodies and lipopeptides for the development of therapeutics and prophylactics in nasal spray and inhalation formulations for the prevention and treatment of COVID-19 and other HCoV infectious diseases.

## Conclusions

In summary, we have designed and constructed a bivalent protein by linking a broad-spectrum antiviral lectin GRFT and a pan-CoV fusion inhibitor EK1 with highly potent inhibitory activity against infection of SARS-CoV-2 and its variants, as well as other HCoVs, including SARS-CoV, MERS-CoV, HCoV-NL63, HCoV-229E and HCoV-OC43. This bivalent, bifunctional recombinant protein can be further developed for prevention and treatment of COVID-19 and other HCoV infectious diseases.

## Data Availability

The datasets presented/analyzed during the current study are available.

## Abbreviations

SARS-CoV-2: Severe acute respiratory syndrome coronavirus-2
SARS-CoV: Severe acute respiratory syndrome coronavirus
MERS-CoV: Middle East respiratory syndrome coronavirus
HCoV: Human coronavirus
GRFT: Griffithsin
IC_50_: Half maximal inhibitory concentration
CC_50_: Half maximal cytotoxicity concentration
ELISA: Enzyme-linked immunosorbent assay
Native-PAGE: Native polyacrylamide gel electrophoresis
6-HB: Six-helix bundle
HR: Heptad repeat
GL15E: Griffithsin-L15-EK1
GL25E: Griffithsin-L25-EK1
GL35E: Griffithsin-L35-EK1
